# Regulation of Lung Epithelial Sodium Channels by Cytokines and Chemokines

**DOI:** 10.3389/fimmu.2017.00766

**Published:** 2017-07-25

**Authors:** Brandi M. Wynne, Li Zou, Valerie Linck, Robert S. Hoover, He-Ping Ma, Douglas C. Eaton

**Affiliations:** ^1^Department of Medicine, Nephrology, Emory University, Atlanta, GA, United States; ^2^Department of Physiology, Emory University, Atlanta, GA, United States; ^3^The Center for Cell and Molecular Signaling, Emory University, Atlanta, GA, United States; ^4^Research Service, Atlanta Veteran’s Administration Medical Center, Decatur, GA, United States

**Keywords:** lung, sodium channels, epithelial sodium channel, cytokines, physiology, inflammation, acute lung injury, acute respiratory distress syndrome

## Abstract

Acute lung injury leading to acute respiratory distress (ARDS) is a global health concern. ARDS patients have significant pulmonary inflammation leading to flooding of the pulmonary alveoli. This prevents normal gas exchange with consequent hypoxemia and causes mortality. A thin fluid layer in the alveoli is normal. The maintenance of this thin layer results from fluid movement out of the pulmonary capillaries into the alveolar interstitium driven by vascular hydrostatic pressure and then through alveolar tight junctions. This is then balanced by fluid reabsorption from the alveolar space mediated by transepithelial salt and water transport through alveolar cells. Reabsorption is a two-step process: first, sodium enters *via* sodium-permeable channels in the apical membranes of alveolar type 1 and 2 cells followed by active extrusion of sodium into the interstitium by the basolateral Na^+^, K^+^-ATPase. Anions follow the cationic charge gradient and water follows the salt-induced osmotic gradient. The proximate cause of alveolar flooding is the result of a failure to reabsorb sufficient salt and water or a failure of the tight junctions to prevent excessive movement of fluid from the interstitium to alveolar lumen. Cytokine- and chemokine-induced inflammation can have a particularly profound effect on lung sodium transport since they can alter both ion channel and barrier function. Cytokines and chemokines affect alveolar amiloride-sensitive epithelial sodium channels (ENaCs), which play a crucial role in sodium transport and fluid reabsorption in the lung. This review discusses the regulation of ENaC *via* local and systemic cytokines during inflammatory disease and the effect on lung fluid balance.

## Introduction

**Figure 1:**
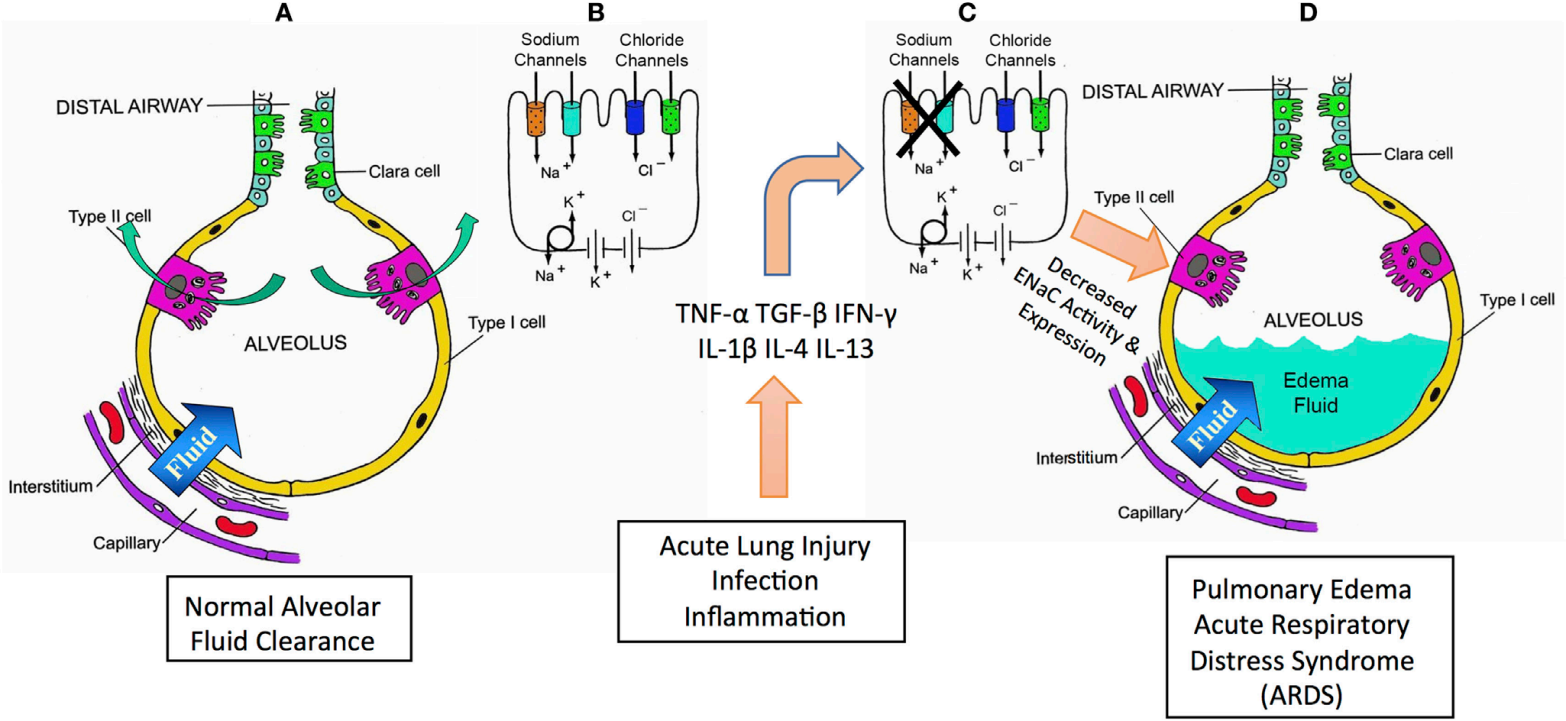
Summary schematic for role of cytokines in mediating pulmonary edema and acute respiratory distress syndrome (ARDS). Acute lung injury (ALI), ARDS, and pneumonia are all pathologies characterized by lung edema and alveolar flooding. Pneumonia mortality is typically caused by flooding of the pulmonary alveoli, preventing normal gas exchange and consequent hypoxemia. Airways normally have a critically regulated fluid layer essential for normal gas exchange and removal of foreign particulates from the airway **(A)**. Maintaining this fluid layer in the alveoli depends critically on sodium reabsorption mediated by epithelial sodium channels (ENaCs) and CFTR chloride channels **(B)**. During ALI, sepsis, inflammation or infection, inflammatory cytokines are produced that inhibit ENaC **(C)**. A decrease in ENaC reabsorption allows fluid to accumulate in the alveoli causing alveolar flood in loss of normal gas exchange and consequent hypoxemia **(D)**.

The maintenance of a thin fluid layer on the surface of the alveolar epithelium is critical for respiration. Two primary mechanisms regulate this fluid layer: Starling’s forces and active sodium (Na^+^) transport. Starling’s forces determine the movement of water from intravascular to extravascular or interstitial spaces caused by hydrostatic and oncotic pressures. An increase in pulmonary vascular pressure accounts for the increased alveolar flooding seen in cardiogenic pulmonary edema. However, the other regulator of the thickness of the alveolar fluid layer is the active transport of Na^+^, followed by potential-driven anion movement through cystic fibrosis transmembrane conductance regulator, and the aquaporin-mediated transport of water. The epithelial sodium channel (ENaC) is critical in the maintenance of the epithelial fluid layer. This review focuses on the primary physiological mechanisms required to maintain and regulate this layer and is an overview of the pathophysiological mechanisms of cytokine-mediated ENaC regulation in the lung (Figure 1).

## Pulmonary Physiology

The primary function of the airways is exchange of gases; thus, both the anatomy and physiology of the lung have evolved to distribute gases efficiently. The diffusion of gases is facilitated in the alveoli by the large total surface area, coupled with thin, yet strong and elastic membranes (1). Human lungs are composed of a series of branched tubes, where conducting airways lead to the terminal respiratory units that are in close proximity to the vasculature (2–4).

The primary respiratory units, or alveoli, are composed of a single, polarized, epithelial cell layer that separates a gas-filled compartment and the pulmonary circulation (5). The two predominant cell types in this cell barrier are the squamous type I (AT1) and cuboidal type 2 (AT2) cells. The majority of the alveolar surface area consists of AT1 cells: the remainder of the area (≈2–5%) is AT2 cells. Both cell types contribute to alveolar fluid transport (6–10). These cells are responsible for Na^+^ transport from the apical to basolateral surface and maintenance of a thin layer of isotonic fluid on the alveolar surface. The AT2 cells have an additional function: they are also responsible for the secretion of surfactant, which is necessary to lower the surface tension at the interface of air and water and increase lung compliance. Overall, this anatomical structure and physiology ensures that the alveolar spaces remain open for gas exchange.

### Paradigm for Fluid Transport: Role of the ENaC

Regulation of the fluid interface occurs primarily through regulating Na^+^ uptake *via* ENaC in both AT1 and AT2 cells. After ENaC-mediated entry of Na^+^ across the apical membrane, Na^+^ leaves the cell across the basolateral membrane *via* the Na^+^–K^+^ ATPase and enters the interstitium where it is in equilibrium with vascular Na^+^. Some investigators have suggested that regulation of the ATPase also plays a role in controlling trans-epithelial Na^+^ transport (11–15); however, we will not consider ATPase regulation in this review. The paradigm in which vectorial Na^+^ transport is considered a primary drive for fluid transport from the alveolar surface has been established by numerous studies where pharmacological inhibitors of apical Na^+^ channels have been shown to reduce the rate at which fluid is cleared (16–21).

### Regulation of ENaC in the Airway

Epithelial sodium channel is composed of three homologous subunits, such as α, β, and γ. Together, these subunits assemble in the endoplasmic reticulum and traffic to the apical membrane and are highly selective for Na^+^ (22). Using ENaCα-subunit knock-out mice, investigators first showed the importance of ENaCα for proper lung function: neonates lacking ENaCα died within 40 h of birth (23). The α subunit is the ionophoric component of the heteromultimer and is required for the expression and assembly of functional ENaCs at the apical membrane. The importance of ENaC to normal lung function is underscored by the phenotype of several monogenetic disorders that affect ENaC. Patients with pseudohypoaldosteronism (PHA), a condition resulting from ENaC partial loss-of-function, were found to have twice the volume of airway surface liquid compared normal levels (24). Mice lacking the ubiquitin ligase, NEDD4-2, had increased levels of ENaC expression and increased ENaC-mediated current in AT2 cells (25). Additionally, overexpression of ENaCβ in an ENaCβ transgenic mouse model leads to airway dehydration and mucous obstruction, comparable to many features observed in cystic fibrosis (CF) (26). Together, these studies highlight the importance of proper ENaC expression and regulation for the airways.

Understanding the regulation of ENaC is significant for understanding lung fluid balance, as ENaC dysregulation is the source of pathological lung edema. In recent years, probably because monogenetic disorders often alter ENaC trafficking, much of the focus has examined how regulation of the number of channels at the apical membrane of alveolar epithelial cells can alter Na^+^ transport. However, since ENaC is an ion channel, regulating how much of the time the channel spends open (the open probability, *P*_o_) is also important. Both Liddle’s syndrome and PHA type 1 (PHA 1) are conventionally described as changes in channel density (an increase and decrease, respectively); however, examination of single ENaCs in these two syndromes shows that ENaC *P*_o_ also changes. There are an observed increase in channel activity in Liddle’s (27) and a decrease in activity in PHA I (28). Steroid hormones increase Na^+^ transport and are often thought to do so by increasing subunit transcription and translation. Although Frindt and Palmer (29) have shown in Na^+^-transporting epithelial tissue that this is indeed true, the increase in subunit density accounts for less than 25% of the increase in trans-epithelial Na^+^ current implying that the remaining 75% is due to an increase in single channel *P*_o_. Single channel recordings show that acute application of steroids dramatically increases single channel *P*_o_ (30–32).

Kleyman, Hughey, and their co-workers have shown that the α and γ subunits of ENaC must be proteolytically cleaved to be active loops (33–37). Some investigators have suggested that such cleavage might be a mechanism by which ENaC in the apical membrane could be regulated. In fact, proteolysis does appear to be required for ENaC to have any appreciable activity, and may be required for it to reach the membrane. As such, proteolysis appears to be, more or less, an all-or-none phenomenon: channels that are uncleaved are capable of little if any activity. However, under conditions of normal Na^+^ transport most channels are cleaved. Under these conditions, cleaved channels are capable of a wide range of activity by changing their *P*_o_.

Changes in membrane ENaC can occur by changing the rate of insertion into the membrane after transcription and translation (38). However, in any time frame less than 24 h, ENaC in the membrane is altered by recycling from intracellular pools into the membrane (22) or internalization of ENaC into recycling or degradative pools. Removal of ENaC occurs primarily *via* the ubiquitin ligase, NEDD 4-2, which targets ENaC for removal and proteosomal degradation (39, 40).

Therefore, in this review, we address both the regulation of *P*_o_ in cleaved channels and change in membrane channel protein density.

The regulation of ENaC occurs *via* multiple, redundant systems to ensure that Na^+^ transport is not limited. ENaC is regulated by a many agents including transmitters interacting with G-protein-coupled receptors (GPCRs), circulating hormones, cytokines and chemokines, and reactive oxygen and nitrogen species. The regulation of ENaC *via* hormones and GPCRs is not a primary focus of this review, but we briefly review ENaC activation and regulation *via* steroids since their actions often interact with the activities of cytokines and chemokines.

In the lung, the glucocorticoid receptor (GR) is the primary receptor for corticosteroids (41–43). Once activated, the GR activates response elements inducing the transcription of signaling kinases, such as the serum- and glucocorticoid-regulated kinase 1 (30, 44, 45). Ligand-mediated activation of the GR *via* corticosteroids is used clinically as an anti-inflammatory treatment. The positive effects of corticosteroid therapy lie in the ability of the GR to bind to and inhibit nuclear factor kappa-light-chain-enhancer of activated B cells (Nf-κB) (46–48). Nf-κB is an important mediator of cytokine signaling. This transcription factor increases cyclooxygenase 2-induced prostaglandin production, as well as increases other proinflammatory factors (49). Corticosteroids reduce inflammation propagated *via* Nf-κB-mediated mechanisms but may not affect inflammation mediated by other signaling pathways. Indeed, GR activation may actually augment some downstream signaling pathways, such as those mediated through Smad proteins (50). This distinction is important because of the many heterogeneous pathways activated by each cytokine (51–54).

## Pulmonary Pathophysiology

Regulation of the air/water interface is crucial for gas exchange, as the amount of the alveolar fluid layer must be precise. With injury, the inability of the lungs to clear this fluid can lead to pulmonary edema. Increased fluid accumulation can result from compromised ENaC function or when there is an asymmetrical hydrostatic force from the vasculature, pushing fluid from capillaries into the alveolar space (e.g., pulmonary hypertension) (5). In addition, tight junctions that maintain structural integrity and a tight epithelial layer can be disrupted, resulting in increased permeability. In fact, high levels of pulmonary inflammation causing airway tight junction damage that compromise alveolar barrier function is a primary cause of epithelial injury (55). And lastly, many proinflammatory and noxious factors cause changes in Na^+^ transport. Dysfunction in any single factor can lead to a dysregulation of the alveolar fluid (5). Because of the increased morbidity and mortality associated with alveolar fluid accumulation, understanding the mechanisms that regulate these factors are vital.

### Immune Responses in the Airway

During inflammation most cells are capable of secreting a variety of small molecular weight proteins, called cytokines and chemokines, which communicate the inflammatory signals. In the airway, resident immune cells are mostly the alveolar macrophages; however, during infection or inflammation, other mononuclear and granular immune cells infiltrate (56). Several studies have proposed a role for the airway epithelium in propagating the immune response, especially as a “first responder” since the airway is the first to sense viral and bacterial pathogens as they enter the body. This layer can be an active participant in the immune response, producing a variety of cytokines and chemokines, as well as exclusive epithelial-derived cytokines (55, 57, 58).

Direct interaction with pathogens, such as influenza, reduces ENaC activity (59). However, other evidence suggests that some of the more chronic effects of pathogens may be *via* noxae-stimulated chronic cytokine production (60–62). Additionally, inflammatory activation of the airway epithelium can result in local nitric oxide (NO) production, most likely *via* increased cytokine production, further reducing ENaC activity and fluid transport (59, 63–67). Cytokines frequently increase local levels of reactive oxygen species (ROS) as well. Interestingly, ROS has been shown to activate ENaC at relatively low concentrations but to inhibit ENaC at higher concentrations often associated with massive pathogen-induced cytokine production (40, 68, 69). The overall redox environment of the alveoli is crucial and can rapidly change, often driven by high levels of Rac1-NADPH oxidase activity in AT1 cells (67, 70).

## Cytokine-Mediated Regulation of ENaC

Some of the earliest studies revealed a correlation between large and sustained proinflammatory cytokine increases in bronchioalveolar lavage (BAL) fluid and an unfavorable outcome in acute respiratory distress syndrome (ARDS) (71). Overall, increased cytokine levels from lung injury can quickly lead to the accumulation of alveolar fluid, edema, and then acute respiratory distress. Thus, proinflammatory cytokines and chemokines produce a feed forward cycle decreasing lung Na^+^ transporter expression, as well as activity.

### Regulation of ENaC *via* Tumor Necrosis Factor (TNF)-α

The TNF super family comprises 19 members and was originally named for its role in apoptosis (53). The best-studied member of this family is TNF-α, which plays a role in propagating the immune response and secretion of other cytokines. TNF-α was implicated in the pathogenesis of pulmonary edema, and increased levels were observed in patients with ARDS (72, 73). Monocytes and macrophages produce significant TNF-α, but it is also produced by alveolar epithelial cells following lipopolysaccharide stimulation (74).

Although TNF-α can bind to two different receptors that are linked to separate signaling pathways, much of the work in the airway has focused on TNF receptor 1. The effect that TNF-α elicits on ENaC function, and alveolar liquid clearance, seems to be critically dependent upon receptor activation or receptor-independent mechanisms and has been shown using both *in vitro* and *in vivo* models (75–78).

Tumor necrosis factor receptor 1-mediated activation of NF-κB increases cytokine (IL-1, IL-8, IL-6) and chemokine production. It also increases the expression of adhesion molecules including selectins, vascular cell adhesion molecules, and intercellular adhesion molecule (ICAM)-1 (53, 79, 80). In freshly isolated AT2 cells, TNF-α decreased α- and γ-ENaC mRNA and protein levels and reduced amiloride-sensitive trans-epithelial current (75, 78).

Tumor necrosis factor-α also plays an especially important role in endothelial activation, as well as disturbing the epithelial tight junction barrier. Disruption of the tight junctions not only leads to respiratory distress and increased exudate but also may reduce alveolar fluid clearance as well (81). TNF-α reduces the expression of tight junction proteins, including the claudins and zonula occludens protein 1, thus increasing alveolar permeability (55, 82). Consequently, TNF-α has a critical and multi-faceted role in the development of ARDS. TNF-α not only regulates Na^+^ and water clearance but also disrupts tight junction barriers and endothelial integrity and contributes to a pro-inflammatory environment.

Interestingly, TNF-α contains not only a receptor-binding domain but also a lectin-like domain (referred to as a TIP domain) that is spatially distinct from the receptor-binding site (83, 84). TNF-α produces an opposite response when there is binding of the lectin-like domain, or TIP, to certain oligosaccharides at high concentrations of TNF-α. This process increases Na^+^ uptake in AT2 cells and may account for the differential responses to TNF-α (85–87). *In vivo*, a peptide analog of TIP increased clearance in a murine flooded-lung model (85). Czikora and colleagues also demonstrated that this TIP domain directly binds to and, then, activates ENaC (83). This implies an endogenous mechanism to limit the effects of high TNF-α concentrations. Use of this may become a novel method in counteracting reduced alveolar clearance.

### Regulation of ENaC *via* Transforming Growth Factor (TGF)-β1

Transforming growth factor-β1 is a pathogenic cytokine, which has been implicated in the early phase of acute lung injury (ALI) prior to ARDS (72, 88). TGF-β1 levels were increased in ARDS patients compared to healthy controls (89). Furthermore, active TGF-β1 levels were more than doubled in the epithelial lining fluid from ARDS patients (90). As mentioned earlier, corticosteroids are a common tool to reduce inflammation and aid in lung clearance. Interestingly, TGF-β actually reduces the ability to produce multiple steroids, possibly leading to the inability for self-healing and furthering inflammatory damage, in addition to the activation of multiple Smad pathways (50, 52). Some of these pathways may be insensitive to corticosteroid treatment. However, there is still much to learn regarding which Smad-mediated pathways are downstream of TGF-β signaling during ALI and ARDS.

Other studies have specifically have explored the role of TGF-β in alveolar flooding. Using a bleomycin-induced lung injury model, TGF-β1-inducible genes were dramatically increased as early as 2 days, suggesting that TGF-β1 may precede alveolar flooding (91). Of interest, TGF-β may actually remain latent locally, covalently attached to a latency-associated peptide (LAP); pulmonary epithelial cells can activate and cause dissociation of TGF-β from LAP (92–94). One member of the integrin family, ανβ6, was recently shown to be a ligand for LAP (93). ανβ6 is expressed normally at lower levels, yet increased significantly with injury revealing a novel mechanism for rapid and local TGF-β activation (95). TGF-β is also redox sensitive, and *in vitro* models of increased ROS *via* ionizing radiation revealed another mechanism for TGF-β activation (96). Together, these studies show multiple, redundant possibilities for systemic and paracrine TGF-β activation during lung injury.

One of the first studies to directly implicate TGF-β1 in regulating ENaC was by Frank and colleagues. They showed that TGF-β1 reduced amiloride-sensitive Na^+^ transport in lung epithelial cells. Additionally, TGF-β1 reduced αENaC mRNA and protein expression *via* an ERK1/2 pathway in a model of ALI, thus promoting alveolar edema (97). *In vivo* studies then showed that TGF-β1 reduces vectorial Na^+^ and water transport and that this process occurs independently from increases in epithelial permeability (97, 98). Interestingly, TGF-β was also found to have an integral role in ENaC trafficking. Peters and colleagues were the first to demonstrate this acute regulation of ENaC in the lung; they found that TGF-β induces ENaC internalization *via* interaction with ENaCβ (99). In summary, TGF-β has been implicated in multiple mechanisms reducing ENaC expression and apical localization, thus contributing to the pathophysiology of ARDS and pulmonary edema (100).

### Regulation of ENaC by Interferon-γ

The interferons (IFN) are a family of proteins originally classified by their ability to reduce viral replication. This family consists of both Type I and Type II IFNs; INF-γ is the only member of the Type II IFN family and is structurally unrelated to the other IFNs. During inflammation, INF-γ is secreted by multiple immune cells, but mostly by T lymphocytes. INF-γ increases ICAM-1 levels and increases NO production *via* inducible nitric oxide synthase. Little is known about the role of INF-γ in ENaC regulation; however, studies using human bronchial epithelial cells (BECs) showed that INF-γ treatment significantly reduced trans-epithelial Na^+^ transport in normal human BECs (101).

### Regulation of ENaC by the Interleukins: IL-1β, IL-4, and IL-13

#### ENaC Regulation by IL-1β

Several interleukins are correlated with the early stages of ALI; however, the best studied is IL-1β. This cytokine plays a diverse role in the pathogenesis of ALI and ARDS. IL-1β levels are increased in the BAL fluid, as well as the pulmonary edema fluid, of patients with ALI (102–106). IL-1β levels are higher in the pulmonary lavage fluids compared to serum suggesting that there is a local, pulmonary source for IL-1β similar to that of TGF-β (71, 104). An earlier study by Pugin and colleagues suggested that of the cytokines present in the BAL fluid, IL-1β is the most biologically active, and others have suggested that the source may be from early-infiltrating neutrophils (103, 107). BAL fluids from ARDS patients applied to AT2 cells increased ICAM-1 expression, while IL-1 inhibition reduced the increase in ICAM-1 (103).

IL-1β also seems to have significant effects on endothelial leakage and permeability. *In vitro*, IL-1β treatment significantly increased microvascular permeability (108). Several studies have also demonstrated that when given intratracheally, IL-1β increased endothelial permeability and lung leak (108–111). More recently, IL-1β has been shown to directly affect ENaC expression. Incubation with IL-1β reduced ENaC mRNA protein expression, possibly through promoter inhibition and a p38 MAPK-dependent mechanism. Additionally, IL-1β application reduced apical ENaC protein and amiloride-sensitive trans-epithelial current and Na^+^ flux (112).

Other studies have tried to reverse IL-1β effects. *In vitro* modeling suggests that the reduction of IL-1β, *via* suppressor of cytokine signaling-1, can rescue the IL-1β-mediated suppression of ENaCs (113). When investigating patients with ALI, those who had an increased activation of the stress protein response (SPR) positively correlated with preserved alveolar clearance rates (114). Thus, activation of this SPR during immune-related injury may ameliorate effects of IL-1β, if used as a “preconditioning” agent (115).

#### ENaC Regulation by IL-4 and IL-13

Classically, increases in IL-4 and IL-13 are associated with an increased goblet-cell hyperplasia and mucous secretion. These cytokines are implicated in allergic airway diseases and CF and contribute to reduced ciliary movement reducing the ability to clear the airways. These related cytokines frequently share signaling cascades and receptor subunits, such as the IL-4 receptor (116). However, studies in airway epithelial cells from human bronchi suggest that these cytokines may also alter ion transport. IL-4 significantly reduced ENaC subunits γ and β; interestingly, αENaC levels were not altered. IL-4 and IL-13 treatments reduced amiloride-sensitive short circuit current (using an Ussing chamber), which was reversed with an IL-4 receptor antagonist (116). Although these studies were investigating allergic diseases, one could infer a similar involvement in a variety of other inflammatory conditions where there are increased IL-4/IL-13 levels and reduced ENaC function.

## Conclusion: Balancing the Inflammatory Milieu

Delineating the role of pro-inflammatory cytokines is important for the understanding of alveolar flooding and ALI; however, the lack of anti-inflammatory cytokines also plays a crucial role in mediating the “balance” necessary for regulating the epithelial fluid lining. Studies have shown that there is an increased mortality when there are reduced levels of “anti-inflammatory” cytokines, such as IL-10 and the IL-1 receptor antagonists (117). Much work is needed to understand the diverse and redundant roles of cytokines in disease progression. Nonetheless, pro-inflammatory cytokines seem to reduce the total expression, apical localization, and activity of ENaC in the lungs *via* multiple mechanisms (Figure 1). Given the prominent role for ENaC in maintaining alveolar fluid levels, understanding how inflammatory cytokines regulate ENaC will allow for the development of therapies to treat these complex diseases.

## Author Contributions

BW and DE conceived and wrote the manuscript. LZ, VL, H-PM, and RH edited and approved the manuscript.

## Conflict of Interest Statement

The authors declare no commercial or financial relationships that could be construed as a potential conflict of interest.
